# Peer-based Education and the Integration of HIV and Sexual and Reproductive Health Services for Young People in Vietnam: Evidence from a Project Evaluation

**DOI:** 10.1371/journal.pone.0080951

**Published:** 2013-11-28

**Authors:** Anh D. Ngo, Toan H. Ha, John Rule, Chinh V. Dang

**Affiliations:** 1 Kolling Institute of Medical Research, University of Sydney, Sydney, Australia; 2 Sydney Medical School, University of Sydney, Sydney, Australia; 3 Independent Consultant, Hanoi, Vietnam; 4 School of Public Health and Community Medicine, University of New South Wales, Sydney, Australia; 5 Ho Chi Minh Institute of Hygiene and Public Health, Ho Chi Minh, Vietnam; 6 Center for Promotion of Advancement of Society (CPAS), Hanoi, Vietnam; George Mason University, United States of America

## Abstract

**Introduction:**

This paper reports changes in behavioral outcomes related to the use of HIV testing service of a project that employed peer-based education strategies and integration of HIV voluntary counseling and testing (VCT) and Sexual and Reproductive Health (SRH) services targeting young people aged 15–24 across 5 provinces in Vietnam.

**Methods:**

A pre-test/post-test, non-experimental evaluation design was used. Data were collected from cross-sectional surveys of youth and client exit interviews at project supported SRH clinics conducted at baseline and again at 24 months following implementation. The baseline samples consisted of 813 youth and 399 exit clients. The end line samples included 501 youths and 399 exit clients. Z test was used to assess changes in behavioral outcomes.

**Results:**

Results show that there was a significant increase (p<0.05) in the percentage of youth who wanted to obtain a HIV test (from 33% to 51%), who had ever had a test (from 7.5% to 15%), and who had a repeat test in the last 12 months (from 54.5% to 67.5%). Exit client interviews found a nearly five-fold increase in the percentage of clients seeking HIV VCT in their current visit (5.0% vs. 24.5%) and almost two-fold increase in the percentage of those having their last test at a project supported clinic (9.3% vs. 17.8%). There were also positive changes in some aspects of youth HIV/AIDS knowledge, attitudes, and risk perceptions.

**Conclusions:**

This study provides preliminary evidence regarding the benefits of the integration of HIV VCT-SRH services in terms of increased access to HIV services and testing in Vietnam. Benefits of peer-based education regarding increased HIV knowledge were also identified. Further investigations, including experimental studies with assessment of health outcomes and the uptake of HIV testing services, are required to better elucidate the effectiveness and challenges of this intervention model in Vietnam.

## Introduction

Access to sexual and reproductive health (SRH) services and access to HIV prevention, treatment, care and support are some of the key targets of the Millennium Development Goals [1]. There has been growing discussion about the potential multiple benefits of linkages between SRH and HIV programs in accelerating the achievements of universal access to SRH services and sustainable interventions for HIV. Since the majority of HIV infections are sexually transmitted or are associated with pregnancy and childbirth [2], then linking HIV and SRH services can prevent risky behaviors that result in HIV infection and adverse reproductive health outcomes (e.g., unwanted pregnancy, unsafe abortion) [3]. Research, worldwide, has documented that integration of HIV testing into SRH services can produce beneficial effects on a number of HIV-related health and behavioral outcomes: reducing HIV and sexually transmitted infections (STIs) incidence, increasing use of condoms and contraceptives, improving coverage and uptake of HIV services including HIV testing, enhancing service quality, and strengthening knowledge of HIV and STIs. Because it reduces the stigma and discrimination associated with attending HIV voluntary counseling and testing (VCT) exclusive services, the integration of services has promoted access to, and uptake of HIV testing (3–5). Service integration also removes redundancies in vertical programs and is able to meet clients’ multiple needs, thus optimizing program effectiveness and efficiency, especially in resource poor settings [4].

Peer-based education interventions have frequently been used as strategies for preventing HIV and other STIs amongst a number of target populations, including youth in developing countries. A meta-analysis of 13 studies reported that peer education interventions were significantly associated with increased HIV knowledge and improved behavioral outcomes [5]. Peer education has also been employed in previous HIV/AIDS interventions as an effective measure to promote STIs treatment seeking behaviors, access to information and utilization of SRH and HIV/AIDS services [6], [7], [8], [9].

The first case of HIV infection was reported in 1990, in Ho Chi Minh city, since then HIV/AIDS has become a significant public health problem in Vietnam (12). Currently, the HIV epidemic in Vietnam is described as concentrated. The 2011 national data estimated that adult HIV prevalence was 0.45% with young people aged 15 –24 accounting for 53.6% of all HIV reported cases [10]. This figure is similar to the worldwide distribution with more than 50% of HIV infection cases being recorded among this age group [11]. Young people have been identified as an important target group in the national HIV prevention and control strategy [12].

In Vietnam, as in other countries, VCT for HIV constitutes a major component of the national strategy for HIV prevention and control [12], [13], [14], [15]. Access to centers providing exclusive VCT services have been limited [16], [17], [18], most likely due to stigma associated with HIV testing, the lack of accompanied medical services, and inadequate social service support for those who test positive for HIV [19]. Recognizing the importance of SRH-HIV services integration, the national guidelines on SRH-HIV linkage in health facilities is being developed. To date, however, no studies have examined the benefits of this intervention strategy to support the current development of the national guidelines in Vietnam.

This study based on an analysis of baseline and end line survey data from the first project in Vietnam that employed the integration of VCT into existing SRH services to target young people aged 15–24. The study aimed to document changes in HIV knowledge, attitudes, and behaviors, and changes in the use of HIV testing services in the target population. We hypothesized that as a result of the project interventions, there would be significant increases in respondents’ HIV knowledge, attitudes, and behaviors and significant increases in their use of HIV testing services.

## Methods

The research protocol and consent procedure were reviewed and approved by MSIV. Ethical approval including approval of consent procedure was provided by the Ethic Panel of the Provincial Department of Health in each province, namely, Hanoi, Nghe An, Ha Tinh, Ho Chi Minh city, and Binh Duong. Written consent was obtained from participants aged 18 years or older, or from parents of participants under 18 years of age after a clear explanation of the study objectives and potential benefits and risks. Participants were also given the opportunity to withdraw from the study at any time. Confidentiality was strictly protected using personal identification codes and data files were protected by password.

### Setting and intervention description

The project was implemented from 2007–2009 by Maries Stopes International Vietnam (MSIV) in partnership with the provincial department of health. The project covered five provinces and cities where MSIV SRH centers were located: Hanoi (northern Vietnam), Nghe An and Ha Tinh (central Vietnam), Ho Chi Minh city and Binh Duong (southern Vietnam). For the first time, young people, aged 15–24 in communities and industrial zones were targeted in the integration of VCT into SRH services. The project was expected to achieve three aims: promote access to friendly and effective VCT services; influence clients’ HIV risk behaviors; and facilitate emotional, social and medical support for clients who tested positive for HIV.

In efforts to develop a culturally appropriate VCT service model, district and facility level situational analyses were conducted at each MSIV SRH center site before implementation. A series of focus group discussions with youth and key informant interviews with community representatives, local health authorities, clinic clients and service providers were held. Focus groups and interviews provided an in-depth understanding of the epidemiological, cultural, behavioral and economic contexts of the project and helped identify potential client groups for VCT services. In addition, youth and local communities were given opportunities to provide input to the development of project activities and materials. The involvement of youth and communities aimed to ensure that interventions were culturally and contextually relevant and that integrated services were tailored to meet client specific needs.

In each province, the project supported the MSIV SRH center and two existing government SRH clinics in urban, sub-urban, and rural areas. In Binh Duong province in particular, the selected clinics were located in an industrial zone and intended to reach factory workers. While the MSIV SRH center was an independent center, the government SRH clinic was part of the district health center. Both types of these clinic facilities were established prior to the project to offer SRH and family planning services (e.g., gynecological or STI check-up, family planning, antenatal care, or SRH counseling) for young people and general populations in the catchment areas. Before the intervention, HIV testing services were only available at the MSIV affiliated center.

Interventions at clinic level consisted of training on HIV VCT for service providers, upgrading clinic facilities, and the provision of HIV test kits. Initially, the project technical team, staffed by a project manager, an advocacy specialist, a counseling trainer, a community and peer education specialist, a lab technician and HIV testing trainer, was created. The team underwent intensive training on the UNAID/WHO HIV testing protocols delivered by MSIV regional experts. The technical team subsequently provided training to clinic medical staff, counselors, and laboratory technicians at designated clinics.

In each project site, a team of 20 peer-educators were recruited and provided with training and refresher training on outreach and communication skills. Peer educators then conducted outreach activities, distributed IEC materials, and, using a referral card system, referred youth to VCT and SRH services at the project supported clinics in catchments areas. These activities were aimed at increasing availability of information on VCT-SRH integrated services, promoting HIV/AIDS awareness, and linking communities to VCT services**.** Women and men who came to the clinic for SRH services were offered free VCT services.

### Study Design and Data collection

A pre-test/post-test cross-sectional non-experimental design was used for the current study. HIV knowledge and behavioral indicators and HIV testing experience were measured in cross-sectional surveys of youth from communities, or factories in catchment areas where a project supported clinic was located. In addition, client exit interviews were conducted at the MSIV SRH center in each province. Data were collected at baseline prior to implementation of the project (December, 2006 ) and again at 24 months following implementation (January, 2009).

For the survey of community and factory youth, the questionnaire consisted of items assessing respondents’ knowledge, attitudes, and behaviors related to HIV/AIDS, and their past use of HIV VCT services. Respondents were also asked: whether they ever had sex or used alcohol in the last month, whether they knew any of friends currently using or injecting drugs, and whether they had met a peer educator during the previous 12 months. For the exit interviews, clients were asked: if they had been tested for HIV, the health facility attended for their most recent test, and the main reason for their current clinic visit. In all interviews, demographic characteristics of participants (sex, age, marital status, education, occupation), and the duration in which they had been living in the area during the period of project implementation were also recorded.

**Sample size and sampling.** The baseline samples consisted of 813 community and factory youth and 399 exit clients. The end line samples included 501 community and factory youth and 388 exit clients. The end line sample size of community and factory youth was determined based on a statistical power of 80% to detect a HIV testing usage rate of 7.5% – the proportion reported in the baseline survey.

A two staged cluster sampling design was used to recruit a sample of community and factory youth in each project site. In the first stage, residential groups or factories were selected proportional to the size of their population. In the second stage, youth aged 15–24 were randomly recruited for interview conducted at their home (for community youth) or in their workplace (for factory youth) by a trained interviewer.

At each MSIV center clinic, around 80 exit clients were invited to attend a questionnaire-based interview by a trained interviewer. Due to logistic challenges, clients presenting at other clinics supported by the project were not interviewed. Informed consent was obtained prior to each interview. In each province, ethical approval was given by the Ethics Panels of the Provincial Departments of Health.

### Data analysis

Data were entered into the Epi Info then transferred to Stata version 9.0 for processing and analysis. Initially, descriptive statistics were undertaken to provide frequency distribution of participants’ demographic characteristics and the evaluation indicators, adjusted for the cluster sampling design. Following this, frequencies of desired or correct responses to specific questions measured before and after project implementation were compared using z test [20] with the significant level of *p*<0.05. Due to the small sample sizes, we were unable to assess the change separately for each site.

## Results

### Community and factory youth survey

Table 1 presents the distribution of demographic characteristics of youth respondents. Other than the smaller proportion of factory youth in the end line sample (p<0.05*),* the two samples were comparable.

**Table 1:** Demographic characteristics of community and factory youth.

Variable	2006 (n = 813)		2009 (n = 501)	
	n	%	n	%
**Age**				
15–19 years	316	38.8	225	45.0
20–24 years	499	61.2	276	55.0
**Gender**				
Male	383	47	201	42
Female	432	53	299	58
**Marital status**				
Married with spouse	56	6.9	44	8.8
Married living apart	6	0.7	5	1.0
Never married	749	91.9	451	90.2
Widow/divorced	4	0.5	0	0.0
**Education**				
Primary (1–6)	36	4.4	10	2.0
Secondary	702	86.2	487	98.0
Upper high	77	9.4	0	0.0
Average years	10.9		10.7	
**Occupation**				
Housewife	21	2.6	17	3.4
Factory worker	219	26.8	104	20.8*
Post-high school student	424	52.0	289	57.8
High school student	16	2.0	8	1.6
Other	135	16.6	82	16.4
**Mobility**				
Average duration living in the area (years)	12.3		11.7	
Away from home > 1 month last year	112	13.7	12	2.4
Spouse away >1 month last year/n	19/56	34.0	8/44	18.2

* : p*<*0.05.

Table 2 summarizes outcomes measured at baseline and 24 months after project implementation with regards to youth HIV knowledge and behaviors, risk perceptions, and attitudes towards people living with HIV/AIDS (PLWH). Generally, there was a significant increase in the percentage of youth who reported knowing how HIV is transmitted and not transmitted and how to prevent HIV infection. Specifically, a significantly higher proportion of youth in the end line survey (p<0.05) knew that abstinence (51.4% vs. 69%), having sex with one uninfected partner (80.8% vs. 90.2%), and consistent use of condoms when having sex (87.5% vs. 95.5%) are among measures to prevent sexual transmission of HIV. The percentage of youth who considered that a healthy looking person can be HIV positive was not statistically changed. However, the percentage of those who answered correctly to all first four knowledge questions rose significantly from 36.2% to 49.7% ( p<0.05). A similar increasing trend was found in the percentage of youth who believed that they are at risk for HIV infection (23.2% vs. 33%; p<0.05) and that they would refuse sex or use a condom when knowing their sex partner has STI symptoms (72% vs. 90%; p<0.05).

**Table 2:** HIV knowledge, risk perceptions, and attitudes among community and factory youth.

Indicator/Response		2006 (n = 813)		2009 (n = 501)	
		n	%	n	%
**HIV knowledge**					
Not having sex at all	Yes	419	51.4	345	69.0 *
Having sex with 1 uninfected partner	Yes	659	80.8	452	90.2 *
Consistent condom use	Yes	713	87.5	476	95.5 *
Healthy person can be HIV+	Yes	663	81.3	394	78.6
*Combination all 4 above*	*Yes*	294	36.2	249	49.7*
Transmission by oral sex	No	422	52.0	120	23.8 *
Transmission by anal sex	Yes	397	48.7	181	36.3 *
Transmission by mosquito bite	No	557	68.5	373	74.4
Transmission by sharing meal	No	676	83.2	436	86.9
**Risk perceptions**					
Refuse sex/use condom if the partner has STI symptoms	Yes	586	71.9	451	90.0 *
At risk for HIV	Yes	189	23.2	164	33.0 *
**Attitudes towards PLWH**					
Care for relatives with HIV	Yes	529	64.9	417	83.2 *
Buy food from HIV+ food seller	Yes	265	32.5	228	45.5 *
HIV+ teacher can teach	Yes	540	66.3	393	78.4 *
Should inform spouse/partner if HIV+	Yes	725	89.0	484	96.6 *

* : p<0.05.

Regarding knowledge on the mode of HIV transmission, there was a significant decline (p<0.05) in the proportion of youth who understood that HIV cannot be transmitted via oral sex (52% vs. 23.8%) and who knew that HIV can be transmitted through unprotected anal sex (48.7% vs. 36.3%). There was no significant change, however, in the proportion of youth who gave correct responses to questions on whether mosquito bite or sharing meal can transmit HIV.

Regarding attitudes towards PLWH, there was a significant increase (p<0.05) in the percentage of youth who reported that they would care for their relatives should they be infected with HIV (64.9% vs. 83.2%) and would buy food from a food seller living with HIV/AIDS (32.5% vs. 45.5%). A rising trend was also found in the proportion of youth who thought that a teacher infected with HIV can teach (66.3% vs. 78.4%) and that a person should inform their spouse or partner if he/she is infected with HIV (89% vs. 96.6%) (p<0.05).

Table 3 presents the change in respondents’ attitudes towards and experiences of HIV testing. There was a significant increase (p<0.05) in the percentage of youth who wanted to obtain a HIV test (from 33% to 51%), who had ever had a HIV test (from 7.5% to 15.4%), and who had a repeat test in the previous 12 months (from 54.5% to 67.5%). The proportion of those who reported a willingness to pay for a test also rose significantly from 68.7% to 80.2% (p<0.05). Regarding the health facility where clients sought recent HIV testing services, five HIV test users in the end line sample selected a MSIV clinic compared to one at baseline (p<0.05), while the proportion of those who chose a private VCT clinic or a public VCT center declined (p>0.05). In line with the above positive changes, the proportion of youth who reported meeting a peer educator during the previous 12 months increased significantly from 49.4% to 63.1% (p<0.05).

**Table 3:** HIV testing attitudes and experience, and HIV-related risk behaviors of community and factory youth.

Indicator		2006 (n = 813)		2009 (n = 501)	
		n	%	n	%
**Attitudes towards HIV testing**					
Want to obtain a HIV test		267	32.8	256	51.1 *
Be willing to pay for the test		560	68.7	402	80.2*
Ever have a test		61	7.5	77	15.4*
Had a repeat test in the previous 12 months		33	54.5	52	67.5*
Know the results of the last test		52	94.5	76	98.7
**Location the last test**					
Private VCT clinic		11	18.5	7	9.1
MSIV clinic		1	1.9	5	6.5*
Public VCT center		45	74.0	33	42.8
Other		4	5.6	32	41.6 *
**HIV related risk behaviors**					
Ever had sex		99	12.1	76	15.2
Median age first sex		20 yrs		20 yrs	
Mean # of partners in last 12 months		1.3		1.2	
Alcohol use last month:					
None		536	65.8	270	53.9
1–2 per week		216	26.5	201	40.1
Daily		3	0.4	7	1.4
Knowing a friend using drugs		67	8.6	60	12.0
Knowing a friend injecting drugs in last 3 months		20	2.4	13	2.6
**Meeting a peer educator in last 12 months**		403	49.4	315	63.1 *

* : p< 0.05.

Table 3 also presents frequency distribution of HIV-related behaviors in the two surveys. Overall, only a small proportion of youth reported high HIV risk behaviors in terms of sexual experience, alcohol use, and drug use, and reported risk behaviors of friends. These proportions did not change statistically over the implementation period.

### Client exit interviews

Two samples were not statistically different in terms of socio-demographic characteristics of respondents (age, education, occupation, and mobility) except that there was a smaller number of married men and women currently living with spouse in the end line sample (Table 4).

**Table 4:** Demographic characteristics of exit clients.

Variables	2006 (n = 399)		2009 (n = 388)	
	n	%	n	%
**Age**				
Under 15 years	1	0.3	0	0.0
15 – 19 years	12	3.0	15	3.9
20 – 24 years	140	35.1	146	37.6
25 – 49 years	239	59.9	218	56.2
50 years or more	7	1.7	9	2.3
**Marital status**				
Married with spouse	287	71.9	242	62.5*
Married living apart	6	1.5	16	4.1
Never married	98	24.6	123	31.8
Widow/divorced	8	2.0	6	1.6
**Education**				
Illiterate	0	0.0	0	0.0
1 to 6 years	26	6.5	13	3.8
7+ years	373	93.5	332	96.2
**Occupation**				
Housewife	63	15.8	87	22.5
Factory worker	109	27.3	90	23.3
Post-high school student	54	13.5	60	15.5
High school student	11	2.8	7	1.8
Other	162	40.6	143	36.9

Table 5 presents data on clinic service use, HIV testing experience, and the health facility where clients had the last test prior to the current visit. It appeared that VCT had become a more important service at the MSIV SRH center clinic, evident in a five-fold increase in the percentage of clients who cited this service as the main reason for their current visit (5% vs. 24.5%; p<0.05) and a two-fold increase in the proportion of those who chose the MSIV clinic for their most recent HIV test (9.3% vs. 17.8%; p<0.05). On the other hand, there was no statistical evidence (p>0.05) indicating an increase (or decrease) in the use of SRH services (i.e., family planning, STI check ups and treatment, antenatal care) as the main reason for the current visit. No statistically significant change was found in the proportion of users having the last HIV test at a private VCT clinic or a public VCT center that was not supported by the project. There was a decline, though not statistically significant in the percentage of clients who had been tested for HIV prior to the current clinic visit (25.4% vs. 18.8%; p>0.05). However, a rising trend was found in the percentage of test users who knew the results of the last test (88% vs. 95%; p<0.05).

**Table 5:** HIV testing attitudes and experience of exit clients.

Indicator	2006 (n = 399)		2009 (n = 388)	
	n	%	n	%
**The main reason for the visit**				
Family planning	126	31.6	121	31.3
STIs check up	101	25.3	92	23.8
Antenatal care	40	10.0	46	11.9
HIV VCT	20	5.0	95	24.5*
Other	112	28.1	33	8.5*
**HIV test experience**				
Ever have a test	101	25.4	73	18.8
Know the last test result	89	88.2	71	94.7*
**Facility attended for the last test**				
Private VCT clinic	12	12.2	10	13.7
MSIV clinic	10	9.3	13	17.8*
Public VCT center	77	76.5	49	67.1
Other facilities	2	2.0	1	1.4

* *p*< 0.05.

## Discussion

This study presents the first ever findings on behavioral outcomes of a HIV VCT-SRH integrated intervention project targeting young people in Vietnam. Overall, statistical analyses indicate that almost all baseline indicators were changed in the desired direction after two year project implementation. Notably, there was statistical evidence of increased use of and greater demand for HIV testing services. This finding is consistent with a systematic review that documented positive effects of the HIV VCT-SRH integration on the uptake of HIV testing [21]. Our observations of a positive change in: self-reported services usage, willingness to test for HIV, and some aspects of HIV/AIDS related knowledge, are also similar to the results observed in previous interventions that integrated HIV VCT-SRH services in China [22] and Thailand [23].

The positive change was reflected in the increase in self-reported usage rates for HIV testing (from 7.5% to 15.4%, p<0.05) and usage rates in the last 12 months (from 54.5% to 67.5%; p<0.05) in community and factory youth. In addition, the proportion of youth respondents who reported wanting a HIV test also significantly increased (from 33% to 51%, p<0.05). These changes were also in line with data collected from exit client interviews that showed growing use of VCT services at the MSIV clinic as described earlier. As a result of services integration, the MSIV SRH center would have attracted more clients presenting for HIV VCT services compared to other health facilities (e.g., private VCT clinic, public VCT center) where SRH services may not be available. Furthermore, the increase in self-reported usage rates was also consistent with the increased proportion of youth who reported meeting a peer educator in last 12 months, suggesting that peer support and referral through the use of referral cards would have been effective. It is appropriate to note that given the higher proportion of clients seeking a HIV test in their current visit, the absence of a significant increase in the proportion of those who were previously tested for HIV was not surprising.

In line with the increased proportion of youth who reported meeting a peer educator in the last 12 months, our analysis also revealed changes in youth HIV/AIDS knowledge, attitudes, and risk perceptions. There was a significant increase in the percentage of youth who were able to identify measures to prevent sexual transmission of HIV in terms of abstinence, faithfulness, and consistent use of condoms, who believed that they are at risk for HIV infection, and who reported that they would refuse sex or use a condom if knowing their sex partners have STI symptoms. In particular, the proportion of youth in the end line survey who agreed that abstinence and faithfulness are measures to prevent HIV was higher than the similar statistics reported in the national survey of Vietnamese young people conducted in the same year (69% vs. 35% for not having sex; and 90% vs. 75% for having sex with only one uninfected partner) [24]. More youth reported non-stigmatized attitudes towards PLWH (e.g., caring for relatives with HIV, buying food from a HIV infected person, and believing that a teacher with HIV can teach). Thus, efforts to improve youth HIV knowledge and attitudes and to reduce stigma and discrimination against PLWH through peer-based outreach activities could have produced positive impacts after two year project implementation.

A lack of positive changes in several indicators should be noted. The proportion of youth respondents who reported correct knowledge regarding the risk of HIV transmission via unprotected anal sex and oral sex significantly declined between 2006 and 2009. Additionally, no change was found in the proportion of youth who gave correct responses to questions on whether mosquito bite or sharing meal with a HIV positive person can transmit HIV. It appeared that uncertainty, misunderstanding, and inadequate knowledge with regards to how HIV is transmitted and not transmitted remained common in young people in the project sites. Such deficiencies in youth HIV knowledge suggested that communication strategies employed by the project, particularly the implementation of peer-based education, had not achieved the optimal outcomes. In a systematic review, Medley et al. (2009) documented a number of implementation factors that can contribute to the effectiveness of this intervention strategy including recruitment, training and supervision, retention, and compensation of peer educators [5]. For example, one time training was associated with increased HIV knowledge, while refresher training was not associated with change in HIV knowledge. Hence, the implementation of peer education interventions by the project may require adjustments taking into account these factors to specifically address youth knowledge gaps. In future interventions, emphasis should be placed on correcting youth misperceptions about HIV transmission identified in the present analysis.

In a review of available studies on HIV-SRH linkages, Kennedy et al. 2010 [21] identified several factors that contribute to successful integration of SRH and HIV services: involvement of the community during planning and implementation, ongoing capacity building, positive staff attitudes, non-stigmatizing services, and engagement of target populations. It appeared that these factors were well observed in the current project in at least three ways. Firstly, the intervention was developed building on thorough situational analyses involving youth, communities, VCT clients and service providers who provided intensive input to the design of project activities and materials. Secondly, efforts to engage young people in the project implementation through peer outreach communication activities and community education events would have increased availability of information on VCT services. Finally, intensive and ongoing capacity building undertaken through training support delivered by the project technical team could have improved service providers’ knowledge, attitudes, and technical skills, which in turn, enhanced service quality and attracted more clients to the project supported clinics. This, along with providers’ efforts to facilitate emotional, social and medical support for clients who test positive for HIV, would have reduced stigma and discrimination associated with HIV and HIV testing. All of these factors could explain the positive changes in HIV VCT service use and HIV knowledge in the target population after two year project implementation.

Study findings should be interpreted in conjunction with its limitations. First, baseline and follow-up samples were not entirely comparable, and there were a lack of a control group and incomplete measurement of respondents’ exposure to the project interventions. As such, the observed changes in youth knowledge and behaviors might be susceptible to confounding bias. For example, the changes in some behavioral indicators might be the result of secular trends or other interventions ongoing in the region during the course of project implementation. However, as noted above, the project interventions entailed essential ingredients of a successful HIV-SRH integration. Moreover, some HIV knowledge indicators measured after project implementation were higher than the national survey data. Even without a control group, based on data, it is likely that the proposed association of improvements in youth’s HIV/AIDS knowledge, attitudes, and their use of HIV testing service with the project interventions is plausible.

Second, data on the use of HIV VCT services was self-reported, and was not supplemented with clinic-level data, so it was impossible to assess the impact of intervention on the actual provision of HIV testing services. However, data collected from youth surveys and client exit interviews consistently indicate a rising trend in the use of HIV testing service, providing evidence of the intervention impact. Third, the measures of HIV knowledge (e.g., knowledge on vaginal sex, drug use, and maternal to child transmission) or behavior (e.g., condom use) were incomplete. The evaluation based on available data, therefore, did not entirely reflect potential effects of the project interventions. Fourth, client interviews were only conducted at the MSIV center clinics, results therefore may not be generalized to other clinics that were also supported by the project. Finally, due to the small sample sizes, the analysis was under-powered to evaluate the change separately for each evaluation site. As the sample of exit clients contained all age groups, we were unable to assess the change in the use of HIV VCT services among youth clients separately. Thus, the possibility of increased use of VCT services among youth clients must await future studies for confirmation.

## Conclusions

In the absence of empirical research (e.g., experimental study), this study provides preliminary evidence regarding the benefits of HIV VCT-SRH integrated services in promoting access to HIV services and HIV testing in Vietnamese youth. Furthermore, the documentation of project interventions that entailed essential elements of the successful integration offers complementary evidence to support the current development of the national guidelines on SRH-HIV linkage in health facilities. Study limitations call for further investigations, including experimental studies, to better delineate the effectiveness and challenges of this intervention model in Vietnamese contexts. Future studies should assess clinic-level indicators (e.g., services quality, client volumes, the number of services provided), health outcomes (e.g., STIs incidence), and uptake of HIV testing. Larger sample sizes will also be required to evaluate the intervention impact in each project site. Obviously, replication of the intervention and associated outcomes in any future study would increase confidence in positive changes found in the present analysis.
